# Opportunities and challenges of integrating artificial intelligence in China's elderly care services

**DOI:** 10.1038/s41598-024-60067-w

**Published:** 2024-04-22

**Authors:** Yongyan Zhao, Jian Li

**Affiliations:** 1 School of Humanities and Law, Harbin University, Heilongjiang, 150086 China; 2 School of Innovation and Entrepreneurship, Harbin University, Heilongjiang, 150086 China

**Keywords:** Artificial intelligence, Social pension, Service system, Service evaluation, Community, Health services, Weight management

## Abstract

The challenge of elderly care presents a formidable task, demanding the collective attention of governmental bodies and diverse sectors of society. The integration of Artificial Intelligence (AI) into the research and development of Social Elderly Care Service (ECS) has emerged as a dominant trend, holding substantial importance in the establishment of an efficient ECS system. This study aims to serve as a comprehensive reference for the advancement of China's ECS system, achieved through the harmonious integration of a social ECS system with AI capabilities. This paper introduces the fundamental theory of AI, delving into the intricacies of the greyscale model of AI. Furthermore, it provides an overview of the current landscape of elderly care and elder care institutions, offering scientific data and insights to propel further research on AI development and system construction. Through an analysis of the existing research status, the study identifies prevalent issues within the AI-ECS integration, emphasizing pivotal factors influencing the construction of a robust social ECS system. To address these concerns, the study puts forth specific and viable policy recommendations. Notably, the questionnaire's statistics underscore that 83% of the elderly populace would opt for AI-driven solutions in selecting intelligent products, thereby underscoring the pivotal role of AI within the social ECS system. The challenges facing elderly care systems, including demographic shifts, resource constraints, and evolving societal norms, demand innovative solutions for providing efficient and effective care. This study addresses these challenges by exploring the integration of Artificial Intelligence (AI) into Social Elderly Care Services (ECS) in China. By delving into the theory of AI and assessing the existing research status, the study identifies key issues in AI-ECS integration and proposes viable policy recommendations. Insights from stakeholder surveys further highlight the importance of AI-driven solutions in meeting the needs of the elderly population.

## Introduction

As China's economy rapidly develops, the issue of providing adequate care for its elderly population has become increasingly prominent. Currently, the "home care, regional care, and facility care" model as exemplified by the ECS system has been implemented, and 9073 and 9064 models have been introduced in various counties and cities^1^. The 9073 and 9064 models mean that 90% of elderly people live in their own houses independently, 7% receive care from community and 3% are in nursing homes. Similarly for 9064 models. However, with the sheer number of elderly individuals and the overwhelming social demand for quality care, these three pension models have proven insufficient. As a result, the majority of China's current pension institutions are unable to meet current needs. In light of this, it is urgent to combine AI technology and pension technology in an organic manner and establish an AI-based pension service system that can better address the needs of social development—a new trend in pension service development for the future.

The significance of socially based elder care services (ECS) in addressing the challenges posed by the aging population cannot be emphasized enough^2^. To gain a profound understanding of the foundational ECS system, which encompasses policy support, network establishment, platform utilization, evaluation, and supervision, it is crucial to conduct a comprehensive examination. By delving into the interconnections and practical application of the underlying urban ECS theory, we can enhance our insights into this vital aspect of societal well-being.

Moreover, the resolution of challenges associated with social pension provision extends beyond merely alleviating the burden of societal and economic pension obligations. It holds the potential to liberate resources that can be redirected towards increased investment in national economic development. This, in turn, contributes to fortifying the economic framework of the local market, a pivotal factor for the successful realization of urban–rural integration^3,4^. In essence, socially oriented elder care services not only cater to the immediate needs of the aging population but also play a crucial role in shaping a more sustainable and dynamic economic landscape.

This study examines the socialization of local elder care services (ECS) against the backdrop of the regional social landscape. It offers practical and effective recommendations and countermeasures to address existing problems in the socialization of ECS in Area A. The article's contributions are twofold: (1) The survey and statistical analysis of the willingness of rural elders to participate in social ECS is both authentic and objective, and can furnish valuable empirical evidence for enhancing the social ECS system in rural Heilongjiang Province. (2) Through vertical comparison of the various developmental stages of the social basic ECS system, the article provides a comprehensive overview of the multifaceted changes that have transpired in the ECS system over time.

The study's contributions include acknowledging the significant challenge of elderly care, recognizing AI's growing role in Social Elderly Care Service (ECS) development, aiming to provide a comprehensive AI-integrated guide for China's ECS system, introducing fundamental AI theory, detailing the specific "greyscale model" of AI, presenting a snapshot of the current elderly care landscape, analyzing research status and identifying integration problems, highlighting influential factors for system construction, proposing practical policy recommendations for AI-ECS integration challenges, and showcasing strong elderly preference (83%) for AI-driven elderly care solutions. A greyscale model refers to an image representation that only includes shades of grey, ranging from black to white, without incorporating colors. In this model, each pixel in the image is assigned a shade of grey based on its intensity, with darker shades representing lower intensity and lighter shades representing higher intensity. Greyscale images are commonly used in various applications, including photography, medical imaging, and computer vision, where color information may not be essential, and representing images in a simpler form can be more practical for certain purposes.

This study lies in its comprehensive exploration of the integration of Artificial Intelligence (AI) into China's Social Elderly Care Service (ECS) system. Focusing on the greyscale model of AI, the research provides a thorough analysis of the current landscape of elderly care, elder care institutions, and the potential impact of AI. The study identifies key challenges and issues in the AI-ECS integration and offers specific policy recommendations. A significant finding is that 83% of the elderly population expresses a preference for AI-driven solutions, highlighting the crucial role AI can play in enhancing the efficiency and effectiveness of the social ECS system. This research aims to serve as a valuable reference for advancing China's ECS system through the harmonious incorporation of AI capabilities.

The rest of paper is organized as follows: Secton “Related works” presents the relationship with the existing literature, while Section “AI to promote social elderly care services” discusses the use of AI to promote social elderly care services. In Section “Experiment and analysis of community elderly care service system”, we delve into the Experiment and Analysis of the Community Elderly Care Service System, and finally, Section “Conclusions” provides the conclusion of this work.

## Related works

Currently, the predominant area of research in the field of elderly care pertains to social pension services. As per the findings of Peng and Su^5^, China has entered a phase of moderate aging. This demographic shift necessitates the prompt establishment and enhancement of the elderly care system to foster the high-quality and equitable development of elderly services. In the light of the national strategy aimed at actively responding to the aging population, it is crucial to break free from traditional research boundaries, integrate the life cycle, and amalgamate various disciplines. This approach can facilitate the efficient matching of cloud server supply and demand by precisely identifying effective demand and scientifically dismantling service supply. Leveraging the governance advantages of the "one core and many" approach can yield a high-quality cloud service system led by the Party committee and government, with social participation and national action. It is essential to focus on consolidating local experiences and transforming "Chinese characteristics" into "Chinese advantages," ultimately contributing to the development of "Chinese thinking" and "Chinese solutions" within the ECS system.

Furthermore, Arloto^6^ conducted an analysis of the 3S effect of the FCG burden of the elderly in the southeast of France and identified the factors associated with the change in their burden. The study included non-dependent elderly individuals who received 3S FCG while still living at home. The burden of FCG was evaluated with the Mini-Zarit scale before the establishment of 3S (the first 3S) and six months after the establishment of 3S (the last 3S). The study revealed that the situation of FCG independent of the elderly was comparable to that of the elderly themselves. The integration of 3S into their daily life can help reduce their stress levels.

Furthermore, in a study conducted by Xu and Feng^7^, the impact of social elderly care on various dimensions of life, including life care, spiritual comfort, and economic support, for both urban and rural residents within the context of family elderly care was empirically examined. This investigation utilized the logistic regression model and multiple linear regression model, drawing upon data from the China Health and Elderly Care Follow-up Survey (CHARLS) in 2013 and 2015.

The study's findings revealed nuanced effects of social pension on family pension, encompassing both crowding-out and crowding-in effects. The crowding-out effect predominantly manifested in its impact on life care within family pension, while the participation in social pension programs exhibited positive influences on the spiritual comfort and economic support aspects of family pension. The crowding-in effect, on the other hand, was primarily observed in the positive impact of community medical services on the spiritual comfort and economic support within family support for the elderly, along with the influence of social spiritual life on economic support.

Importantly, the study highlighted substantial variations in the impact of social pension on family pension between urban and rural areas. While rural community medical services demonstrated a positive impact on spiritual comfort and economic support, this effect was less pronounced in urban and town settings, underscoring the existing urban–rural disparity in medical resource distribution. This comprehensive analysis sheds light on the intricate dynamics of social elderly care and its varied implications for family pension in different regions.

China's old-age security system has gone through three stages: national welfare supply, social multi-agent supply, and market diversification supply. To cater to the diverse ECS needs of various income groups, such as home-based elderly care and institutional elderly care for healthy, semi-disabled, and disabled elderly individuals, a multi-level ECS supply system with self-sufficiency, welfare, non-profit, and profitability features has been gradually established under state leadership and administrative promotion. In light of the structural contradiction between insufficient effective demand and insufficient effective supply of ECS, Zhang^8^ has proposed the adoption of a market-oriented approach with an emphasis on supply-side reform to address the issue.

The effective implementation of the ECS system necessitates its adherence to the laws of the socialist market economy that embodies distinctive Chinese characteristics. Such compliance requires the development of a robust supply and demand, price, and competition mechanism for ECS. This mechanism should optimize resource allocation through market-based approaches while simultaneously leveraging the government's role in promoting fair competition, fostering a unified market for ECS, and efficiently regulating the business environment through a fair supervision and legal system.

The intersection between neuroscience and artificial intelligence has been the subject of extensive research. However, recent studies suggest that communication and cooperation between these two fields have waned. In a bibliometric analysis of 2078 articles related to AI and neuroscience, Chen^9^ found that research concentrated on cognitive science and computational neuroscience, cognitive neuroscience, neuroimaging, natural language processing, diagnosis and treatment of nervous system diseases, and humanoid robots. Cross-disciplinary research between AI and neuroscience is on the rise, and future studies may emphasize deep learning, machine learning, functional magnetic resonance imaging, EEG, computational neuroscience, neuroimaging, and classification algorithms.

Despite the growing popularity of artificial intelligence, there remains a need to explore how users evaluate algorithms and their perception of algorithmic decisions and their relationship with algorithm functionality. Shin^10^ conceptualizes the core structure of algorithms that drive embodied value by citing the concept of embodied cognition and characterizes these factors as trust by examining how these factors influence the user experience of personalized recommendation algorithms. The research results highlight the specific cognitive processes involved in the characteristics of inference algorithms, including fairness, accountability, transparency, and interpretability, as well as their fundamental connections with trust and subsequent behavior.

Ng and Lin's study^11^ leveraged ecologically effective social media data to discern the motivations and associated topics underlying interactions with conversational AI in natural settings. The researchers conducted a survey of Reddit user conversations within the social mediation community of practice, employing the potential Dirichlet assignment method to identify AI-driven virtual assistant and intelligent speaker interactions. The findings revealed diverse conversation topics, encompassing functional satisfaction, hedonic satisfaction, social satisfaction, settings, encountered problems, and device connections, exhibiting significant variations over time. The research suggests that members within the sub-Reddit community share a common motivation to engage with conversational AI. Their collaborative discussions revolve around addressing relevant issues and problem-solving, contributing to improved practices within the field. This study underscores the dynamic nature of conversational AI interactions and their integral role in fostering community engagement and problem-solving.

In^12^, Lee employs deep learning and pre-trained language models based on the transformer structure to classify user sentiments in the context of the "Metaverse." Unlike previous research limited to a single platform, this study incorporates review data from YouTube and the Google Play Store for broader applicability. Utilizing Bidirectional Encoder Representations from Transformers (BERT) and the Robustly optimized BERT approach (RoBERTa) models with a soft voting mechanism, the study achieves a remarkable accuracy of 88.57%. Furthermore, the ensemble model, combining RoBERTa, BERT, and A Lite BERT (ALBERT), attains an impressive area under the curve (AUC) score of 0.9458. These results highlight the efficacy of the RoBERTa model, particularly when integrated into an ensemble, making it applicable for platforms offering metaverse services. The study contributes to the progress of natural language processing in metaverse services, crucial in digital platforms and virtual environments, emphasizing the potential of sentiment analysis through deep learning for enhancing user experiences in the metaverse.

Hollander^13^ discussed the role and possible risks of AI-driven automatic social media accounts in participating in the planning process and broader social welfare. Liengpunsakul^14^ look at sustainable development in China with artificial intelligence (AI). The Chinese government has created aspirational plans for taking the lead globally in AI and sustainable development. China is trailing behind in reaching the Sustainable Development Goals (SDGs) overall, while making success in a number of SDG-related sectors. AI technology can help China make more headway in the SDGs. The article also assesses the impact of AI on sustainable development, both globally and regionally, using the most recent data from 193 nations throughout the world. In general, it is shown that progress toward the SDGs and government AI preparedness are strongly positively correlated. Government AI preparedness is discovered to have a positive correlation with the four pillars of the SDGs: economics, society, environment, and partnerships. According to the evaluation presented in^15^, the development strategies for AI in the USA, Russia, and China all incorporate elements aimed at promoting civic responsibility and the responsible use of AI. The Unified framework of five principles for AI in society, devised by Floridi, serves as a valuable assessment tool to gauge how social responsibility is generally advocated and implemented in national AI policy papers. The authors of the paper advocate for further research in the development of mutually recognizable ethical models for socially beneficial AI. In study^16^, a sophisticated intelligent supply chain for Chinese old-age services is constructed and evaluated. The development of China's endowment service's intelligent supply chain is categorized into policy, economic, social, and technical aspects. These significant categories are further detailed into first-level and second-level indicators. Surveys conducted among managers and staff members associated with old-age services determine the importance and satisfaction of each assessment indicator. The study utilizes reliability analysis and importance-performance analysis to create an IPA quadrant analysis chart of the evaluation index. Kumar et al.^17^ delve into the diverse applications of AI in various medical fields, including radiology, dermatology, haematology, and ophthalmology. The paper conducts a comparative analysis using essential criteria and addresses ethical, legal, trust, and long-term implications of AI in healthcare. Additionally, the challenges of widespread AI integration into medical systems are discussed. This comprehensive exploration highlights the multifaceted applications of AI in healthcare and emphasizes the critical considerations in its ethical and legal dimensions.

Jung et al.^18^ gathered data from Reddit and LexisNexis spanning over four years to assess the performance of six machine learning techniques. These techniques incorporated technical and sentiment indicators, along with post volume, to predict Bitcoin price trends. Extreme Gradient Boosting (XGBoost) demonstrated the highest accuracy at 90.57%, with an impressive area under the receiver operating characteristic curve (AUC) value of 97.48%. The study highlighted the effectiveness of combining sentiment analysis using Valence Aware Dictionary and Sentiment Reasoner (VADER) and 11 technical indicators like moving averages, relative strength index (RSI), and stochastic oscillators for Bitcoin price prediction. The findings suggest that these input features can enhance the accuracy of predicting Bitcoin prices, enabling investors to make more informed decisions about Bitcoin-related investments.

The research aimed to investigate the influence of social media manipulation on online discussions concerning land development and planning issues. To gauge this risk, the paper provided a synthesis of pertinent theories and experiences related to the subject. It delved into the impact of artificial intelligence (AI) on the interests of the Twitter community and put forward relevant recommendations. The findings indicated that, during the land development and planning phases, the use of social media accounts has the potential to amplify the voices of individuals contributing to chaos, thereby expanding their influence. However, existing studies lack a comprehensive exposition of the background of community Elderly Care Services (ECS) and a detailed introduction to the overall functions of AI. Furthermore, the solutions proposed for community elderly care issues require further refinement and development.

## AI to promote social elderly care services

### AI

From a technical perspective, AI technology comprises two distinct but related aspects, namely AI and Augmented Intelligence. AI aims to automate and replace routine human tasks with machines, while Augmented Intelligence aims to support and augment human activities in their daily lives and work^19,20^. The field of AI technology is further divided into two categories: Neural Networks and Robotics. Neural Networks are designed to assist and automate cognitive tasks, while Robotics focus on automating physical tasks. Accordingly, current AI research can be classified into four quadrants.

Over the past 60 years, AI has experienced various ups and downs in its development, from the Turing test to the current state-of-the-art deep learning technologies. Each boom period is characterized by different representative technologies, and currently, deep learning is the most popular AI technology. Deep learning relies on algorithmic models, which are jointly shaped by external data and internal tasks^21^. Data is obtained through various sensory inputs from the external environment, and then stored and processed on cloud platforms. Tasks are instructions issued by humans to adjust variables and minimize biases through intrinsic value functions. For each task, a unique value function must be designed to form a specific model, which is then trained on large amounts of data. In essence, deep learning uses big data to extract rules, which are then applied to perform regression prediction, classification, and clustering of unknown inputs to obtain results.

The Grey Model, first proposed by a professor at Huazhong University of Science and Technology in 1982^22^, has attracted considerable attention from scholars and researchers worldwide and has been widely applied in various domains. A Grey System is defined as a system in which, in a data, some information is known, while other information is unknown^23^.

First-order cumulative generation:

$x^{(0)}$ is original collection, unit is bit. The original non-negative data sequence with variable $x^{(0)}$ as:

1 $$x^{(0)} = [x^{(0)} (1),x^{(0)} (2), \ldots ,x^{(0)} (n)]$$

Then the first-order accumulation of $x^{(0)}$ generates a sequence:

2 $$x^{(1)} = [x^{(1)} (1),x^{(1)} (2), \ldots ,x^{(1)} (n)]$$

In the equation,

3 $$x_{{}}^{(1)} (k) = \sum\limits_{i = 1}^{k} {x^{(0)} (i),k = 1,2}$$

In formula (3), k is constant, a quasi-smooth check for $x^{(0)}$ and a quasi-exponential regularity check for :

4 $$\rho (k) = \frac{{x^{(0)} (k)}}{{x^{(0)} (k - 1)}},k = 2,3$$

If satisfied

5 $$\rho (k) < 1$$

In formula (5), $\rho (k)$ shows a decreasing trend, then $x^{(0)}$ is a quasi-smooth sequence, and $x^{(1)}$ has a quasi-exponential law. Otherwise, perform first-order weakening:

6 $$x^{,(0)} (k) = \frac{1}{n - k + 1}(x(k) + x(k + 1) + \cdots + x(n)),k = 1,2$$

Then,

7 $$x^{(0)} (k) = x^{,(0)} (k)$$

In formula (7), that is, $x^{(0)}$ is replaced by $x^{,(0)}$.

It can be seen from the second step that $x^{(1)}$ has an approximate exponential growth law, so it can be considered that sequence $x^{(1)}$ satisfies the following first-order linear differential equation:

8 $$\frac{{dx^{(1)} }}{dt} + ax^{(1)} = u$$

Solutions have to:

9 $$\left[ \begin{gathered} {\hat{a}} \hfill \\ {\hat{u}} \hfill \\ \end{gathered} \right] = (B^{T} B)^{ - 1} B^{T} Y_{n}$$

$$\left[ \begin{gathered} {\hat{a}} \hfill \\ {\hat{u}} \hfill \\ \end{gathered} \right]$$ is the first order differential equation. Among

10 $$Y_{n} = \left[ \begin{gathered} x^{(0)} (2) \hfill \\ x^{(0)} (3) \hfill \\ \vdots \hfill \\ x^{(0)} (n) \hfill \\ \end{gathered} \right]$$

11 $$B = \left[ {\left. {\begin{array}{*{20}c} { - \frac{1}{2}[x^{{(0)}} (1) + x^{{(0)}} (2)]} & 1 \\ { - \frac{1}{2}[x^{{(1)}} (2) + x^{{(1)}} (3)]} & 1 \\ \vdots & \vdots \\ { - \frac{1}{2}[x^{{(1)}} (n - 1) + x^{{(0)}} (n)]} & 1 \\ \end{array} } \right|} \right]$$

In formula (9), substitute the obtained $\hat{a}$ and $\hat{u}$ into the differential Eq. (8), people have

12 $$\frac{{dx^{(1)} }}{dt} + \hat{a}x^{(1)} = \hat{u}$$

The grey prediction model is established. From the differential Eq. (12), the grey prediction model of the cumulative sequence $x^{(1)}$ can be obtained as:

13 $$\hat{x}^{(1)} (k + 1) = [x^{(1)} (0) - \frac{{\hat{u}}}{{\hat{a}}}]e^{{ - \hat{a}k}} + \frac{{\hat{u}}}{{\hat{a}}},k = 0,1,2$$

If $x^{(1)}$ comes from the sequence obtained by the first order weakening of $x^{(0)}$, it can be seen from Eq. (13) that after the first-order weakening is restored:

14 $$\hat{x}^{(0)} (k + 1) = \hat{x}^{(1)} (k + 1)$$

On the contrary, the cumulative reduction is performed by Eq. (14), and the grey prediction model of $x^{(0)}$ is obtained as:

15 $$\hat{x}^{(0)} (k + 1) = (e^{{ - \hat{a}}} - 1)[x^{(0)} (n) - \frac{{\hat{u}}}{{\hat{a}}}]e^{{\_\hat{a}k}} ,K = 0,1,2$$

Test of grey prediction model:

In formula (15), when $- \hat{a} \le 0.3$, it can be used for medium and long-term forecast; when $0.3 < - \hat{a} \le 0.5$, it can be used for short-term forecast, and it can be used with caution in medium and long term; when $0.5 < - \hat{a} \le 0.8$, the short-term forecast is very cautious; when $0.8 < - \hat{a} \le 1$, the residual correction should be used; when $- \hat{a} > 1$, the grey system prediction model should not be used^24^. Then post-check test, let the residual sequence:

16 $$\varepsilon^{(0)} = (\varepsilon (1),\varepsilon (2), \ldots ,\varepsilon (n)) = (x^{(0)} (1) - \hat{x}^{(0)} (1),x^{(0)} (2) - \hat{x}^{(0)} (2), \ldots ,x^{(0)} (n) - \hat{x}^{(0)} (n))$$

The mean $\overline{\varepsilon }$ and variance of the residuals $S_{\varepsilon }^{2}$ are:

17 $$\overline{\varepsilon } = \frac{1}{n}\sum\limits_{k - 1}^{n} {\varepsilon (k)}$$

18 $$S_{\varepsilon }^{2} = \frac{1}{n}\sum\limits_{k - 1}^{n} {(\varepsilon (k) - } \overline{\varepsilon })^{2}$$

The mean and variance of $x^{(0)}$ are:

19 $$\overline{x} = \frac{1}{n}\sum\limits_{k - 1}^{n} {x^{(0)} (k)}$$

20 $$S_{x}^{2} = \frac{1}{n}\sum\limits_{k - 1}^{n} {(x^{(0)} (k) - \overline{x})^{2} }$$

Then the posterior difference ratio C is:

21 $$C = \frac{{S_{e} }}{{S_{x} }}$$

The small error probability is:

22 $$p = p(\left| {\varepsilon (k) - \overline{\varepsilon }} \right| < 0.6745S_{x} )$$

Among them, the smaller C is, the better; and the larger p is, the better.

### Social pension service system

The provision of Social Elderly Care Services (ECS) by family members stands as a fundamental tenet of elderly care within the family unit, constituting a crucial service for the well-being and emotional health of the elderly^25^. In situations where at least one elderly family member is non-disabled and capable of self-support, they often take on the role of primary caregivers, alleviating pressure on other family members and fulfilling the elderly's desire for self-worth^26^. However, this self-supported care model is a temporary solution, as the aging process and changing physical conditions gradually diminish the elderly's ability to provide care for themselves^27^. This inevitability leads to an increasing reliance on others for elderly care, marking a shift from the self-supported model to a more dependent state. Consequently, the traditional family pension model, relying solely on family resources, poses challenges for both elderly family members and their counterparts. Balancing the evolving needs of elderly care within the family structure becomes crucial as individuals age and require more comprehensive support.

#### Demand analysis of ECS

**Figure 1:**
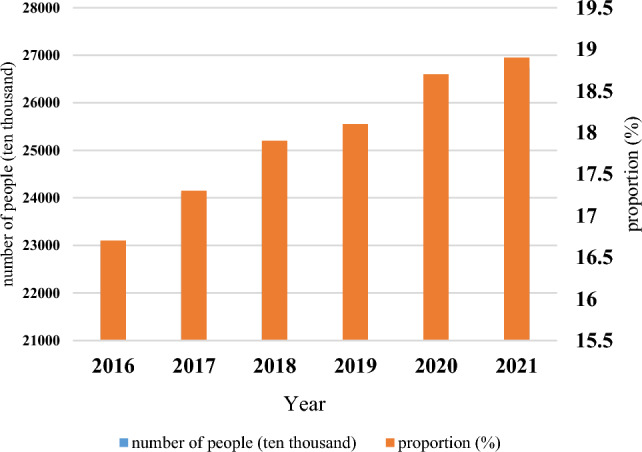
Number and proportion of the population aged 60 and above in China from 2016 to 2021.

Figure 1 displays the population figures for individuals aged 60 years and above in China from 2016 to 2021. By the end of 2021, the elderly population in China had reached 267 million, accounting for 18.9% of the total population. Furthermore, the elderly population aged 65 and above had surpassed 200 million, representing 14.2% of the total population. It is projected that during the 14th Five Year Plan period, the elderly population aged 60 years and above will exceed 300 million, accounting for over 20% of the population, marking a phase of moderate aging. By 2035, the elderly population aged 60 and above is expected to surpass 400 million, accounting for over 30% of the total population, indicative of a severe aging stage^28^, as illustrated in Fig. 1.

Recent analysis of the elderly population in China reveals that the country has a large base and a fast growth rate in terms of aging. This trend is projected to continue into the future. As of 2070, China had the largest elderly population in the world^29^. The severity of China's aging population necessitates a significant scale of pension services in the country.

#### Insufficient social pension service system

**Figure 2:**
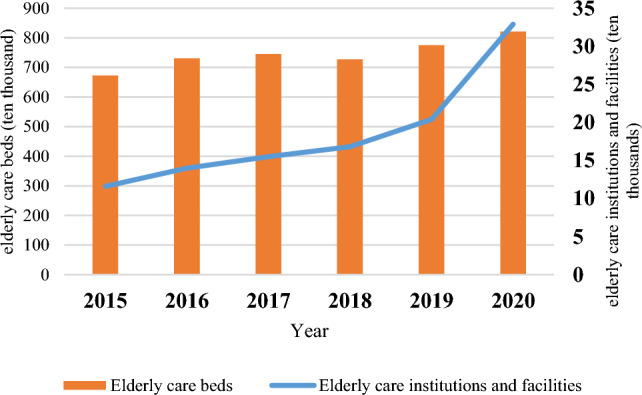
Growing need for elderly care in China: a look at institutions and bed capacity from 2015 to 2020.

Based on the investment and operating entities of pension institutions, China's pension institutions can be classified into four main models^30^. The first model is the public type, wherein the government funds the construction, management, and operation of elderly care institutions, which is a typical model of public-owned elderly care institutions. Initially, this model catered to the elderly with "three noes" and "five guarantees," but it now serves the broader society. Since the government shoulders the funding of public pension institutions, the personnel providing services in such institutions are salaried, and these institutions function as providers of social welfare. The second model is the public-run private type, which is mainly funded by the government and operates through the contracting services of non-governmental organizations. This model is characterized by a separation of ownership and operation rights. Although these institutions retain a certain public welfare nature, their work efficiency surpasses that of public pension institutions. The third model is the private office-assistance type, where non-governmental organizations establish old-age service institutions to provide non-profit services. These institutions are funded mainly by user fees, government subsidies, and social donations. The fourth model is the private type, established through private investment, and provides ECS for profit. Although the government offers certain policy support, such institutions do not rely on the government to sustain their business. Among them, public institutions have greater policy and financial advantages, and their personnel are more stable, the facilities are better, and the fees are lower, resulting in a general shortage of beds. Driven by the market, private elderly care institutions have grown rapidly, but the overall development environment is average, and competition is fierce. If the number is used of beds as an indicator to measure the scale of elderly care institutions, the scale of elderly care institutions in China can be observed in Fig. 2.

As of the close of 2020, the number of registered elderly care institutions nationwide was 38,000, indicating a yearly increase of 4000 and a year-on-year growth of 11.0%. Meanwhile, the number of beds in these institutions stood at 4.882 million, up by 494,000 compared to the preceding year, marking a year-on-year surge of 11.3%. However, an undue emphasis on quantitative growth based on institutional and bed counts may steer elderly care institutions and the society at large to overlook crucial aspects such as the content and quality of elderly care services (ECS)^31^. Thus, in addition to ramping up the number of pension beds, it is imperative to enhance the efficiency of pension services and optimize the utilization rate of existing pension resources to address the shortfall in the scale of elderly care services.

The utilization of elderly care resources in China faces significant challenges, evident in the suboptimal occupancy rates of elderly care institutions. In 2014, the overall vacancy rate for such institutions across the country was notably high, reaching 44.55%. This included a pension vacancy rate of 34.45% for institutional care and an alarming 65.59% for community-based care, Table 1. Despite the low overall occupancy rate, certain institutions operate consistently at full capacity. For instance, the Beijing No. 1 Social Welfare Institute grapples with a waiting list exceeding 10,000 elderly individuals. However, the admission rate is limited, with only a few dozen new residents accepted annually, primarily when existing residents vacate the facility, Table 1. This discrepancy underscores the existing gap in meeting the demand for elderly care services and highlights the need for addressing resource distribution and accessibility challenges within the elderly care system in China.

**Table 1:** The occupancy of China's elderly care institutions in 2014.

	Number of beds (10,000)	Number of people in hospital (10,000 people)	Occupancy rate (%)	Empty bed rate (%)
Nursing home	187. 51	64. 53	34. 41	65. 59
Institutional pension	390.22	255.80	65.55	34.45
Total	577.73	320.33	55.45	44.55

Disregarding the connotative significance of the term "community-based elder care services" (ECS) would inevitably lead to a decline in the richness and caliber of services provided, culminating in an uptick in the rate of unoccupied positions. The aggregate employment of assets intended for endowment purposes within the Chinese domain currently remains at an unsatisfactory level, and a discernible contrast exists between the capacity of social security provision and that required by the aged populace. To optimize the breadth and excellence of service offerings, stakeholders must enhance the efficiency of resource allocation by integrating data concerning supply and demand, harmonizing the distribution of assets, and leveraging information technology^32^.

Given the aforementioned circumstances, this manuscript advocates for the enhancement of the existing medical treatment regimen for geriatric patients by establishing a comprehensive information infrastructure spanning from county-level medical facilities to local communities (i.e., village dwellings). This solution aims to address the issue of information asymmetry, which is currently prevalent in the healthcare system.

**Figure 3:**
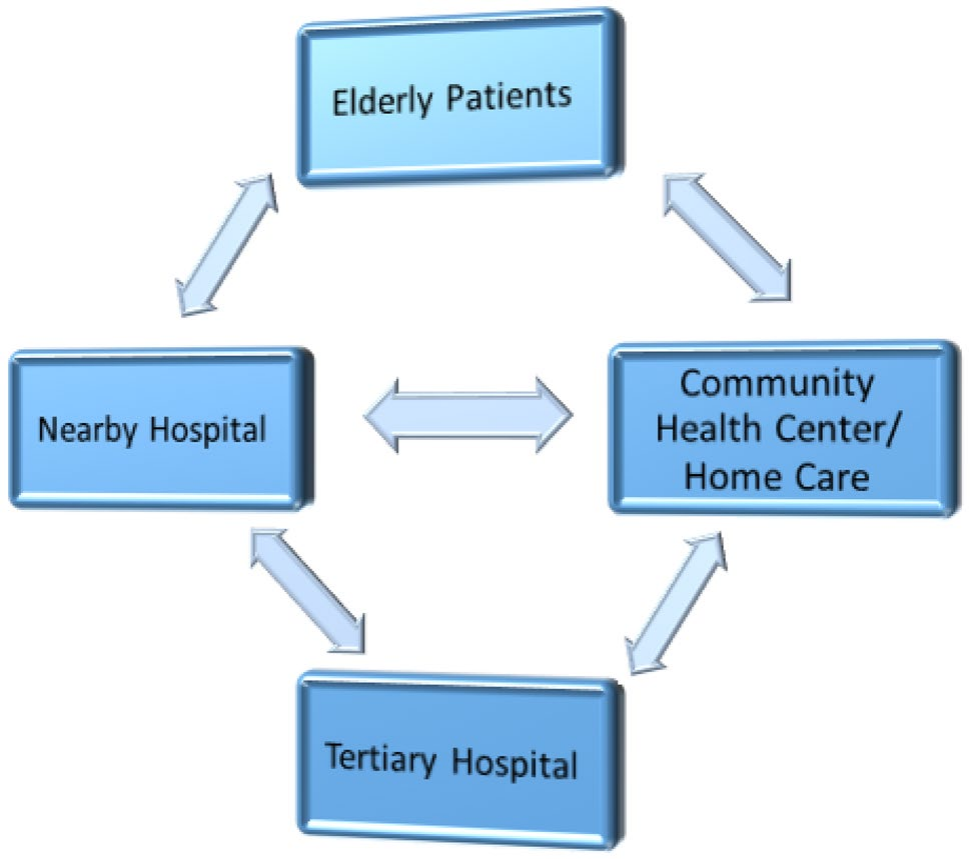
The proposed medical information platform for the elderly.

As depicted in Fig. 3, the first step is to establish an information platform among hospitals to facilitate the exchange of medical treatment information across all levels of hospitals. Each terminal of the information platform is capable of initiating or receiving various types of information such as medical conditions, symptoms, diagnostic reports, treatment suggestions, treatment plans, nursing recommendations, and precautions. Secondly, the terminals of the information platform must be manned by specialized personnel who can upload information such as symptoms and disease progression on behalf of patients or community elderly service practitioners in case of an illness. Full-time doctors in community hospitals can then make a preliminary diagnosis based on the patient's past medical records, assess the severity of the illness, and refer the patient to the appropriate hospital in a timely manner.

Once the patient receives appropriate diagnosis and treatment, the hospital can upload the treatment progress and feedback to the platform, which can be shared with the patient's family or community ECS personnel. The feedback information should include the final diagnosis, treatment plans, medical prescriptions, drug efficacy, physical assessment, nursing recommendations, and other related details. Fourthly, during the recovery stage, the patient's family or community ECS personnel can upload the treatment progress to the platform, enabling the visiting hospital to receive feedback information and plan for follow-up treatment or rehabilitation.

Due to the sharing of medical resources and information, doctors can collaborate and seek medical consultation while customizing health plans for patients. This two-way communication and information exchange can result in timely rescue of patients, efficient use of medical resources, increased doctor-patient communication, and ultimately, avoidance of potential doctor-patient disputes.

### Social pension service system under the background of AI

The integration of cutting-edge AI technologies has ushered in a new era for elderly care services, redefining the quality of care and optimizing the utilization of precious resources dedicated to our senior population. The pivotal role of AI in revolutionizing this domain cannot be overstated. By aligning the offerings of elderly care services (ECS) with the unique needs of the aging population, service providers are empowered to craft bespoke products and solutions. This entails a strategic blend of refining existing resources and introducing innovative service provisions, all made possible through the process of AI-driven advancements.

The advent of Artificial Intelligence (AI) has introduced transformative tools and capabilities, poised to enhance both the efficiency and effectiveness of Elderly Care Services (ECS). This technological integration marks a profound shift in service conceptualization and delivery, heralding an industrial renaissance in elderly care. The emergence of "AI-empowered community home-based care services" exemplifies this transformative change. This paradigm seamlessly integrates innovation pillars like the Internet of Things, big data analytics, and intelligent terminal equipment, meticulously tailored to the nuances of community home-based care.

By synergizing the strengths of institutional and community cloud servers, this innovative approach elevates home cloud servers into dynamic hubs of comprehensive, multi-tiered, efficiency-driven, and user-friendly smart home nursing care. This fusion of online and offline realms nurtures an information ecosystem, catering to diverse daily needs and recreational preferences of the elderly. The outcome is a intricate tapestry of services meticulously optimized to bring boundless joy and satisfaction to the elderly. This signifies a new era in elderly care, where AI empowers a holistic and personalized approach to meet the evolving needs of aging populations.

The synergy between the digital realm and home-based ECS is akin to opening an expansive vista for the elderly, a realm teeming with information, connectivity, and camaraderie. This newfound avenue amplifies their demand for services that incorporate internet-based functionalities into their daily lives, ushering in the era of "internet + home" elderly care. This transformative journey rests upon the solid foundation of big data and technological marvels. By leveraging the omnipresence of network technology, elderly data is seamlessly gathered, stored, and processed, painting an insightful portrait of their needs. This real-time understanding bridges the gap between supply and demand, culminating in a harmonious equilibrium.

As the digital landscape continues its march, enterprises and innovators are keenly harnessing its might to elevate their offerings to new heights. This symbiotic alliance between the digital realm and the elderly community not only shares the societal benefits but also eases the fiscal burden on governments, marking a commendable step towards a robust and elevated quality of elderly care services.

**Figure 4:**
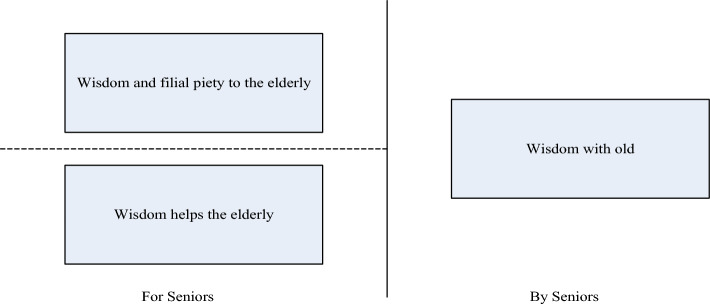
Three dimensions of smart old age care.

The concept of smart elderly care is a trifold marvel: a symphony of wisdom that empowers the elderly, wisdom that enriches the lives of the elderly, and wisdom that pays homage to filial piety. Illustrated in Fig. 4, "For Seniors" on the left signifies our cherished elderly population. This dimension of wisdom envelops them with both material and spiritual support, culminating in a holistic care experience. On the right, "By Seniors" represents the profound reservoir of experience, knowledge, and skills that our elderly possess. The holistic embrace of AI-empowered ECS radically elevates the landscape of elderly care, ushering in an era where technology and compassion seamlessly intertwine, enriching the lives of our senior citizens.

### Evaluation system of social elderly care services

From the perspective of China's administrative management system, a community can be defined as a group of social organizations that are geographically connected and form an autonomous structure at the grassroots level, serving as a fundamental platform for implementing social management. As such, the community serves as a vital component of the national body and acts as the government's representative in providing public goods and services. To achieve the objectives and goals set by the State Council, individuals within the community must work towards improving the modern Elderly Care Service (ECS) system, ensuring its maximum development within the community.

This paper posits that the "eight platforms and six guarantees" system model should be utilized in the development of the community's ECS. The "eight platforms" refer to the essential service categories required by the primary ECS service targets, such as service care, health care, comprehensive assistance, social participation, spiritual care, safety protection, power maintenance, and information management. This must be designed based on comprehensive research and analysis of the specific situation and needs of the elderly within the community. The "six guarantees" constitute the macro environment necessary for the successful implementation of ECS and are established and provided by higher-level government departments.

**Figure 5:**
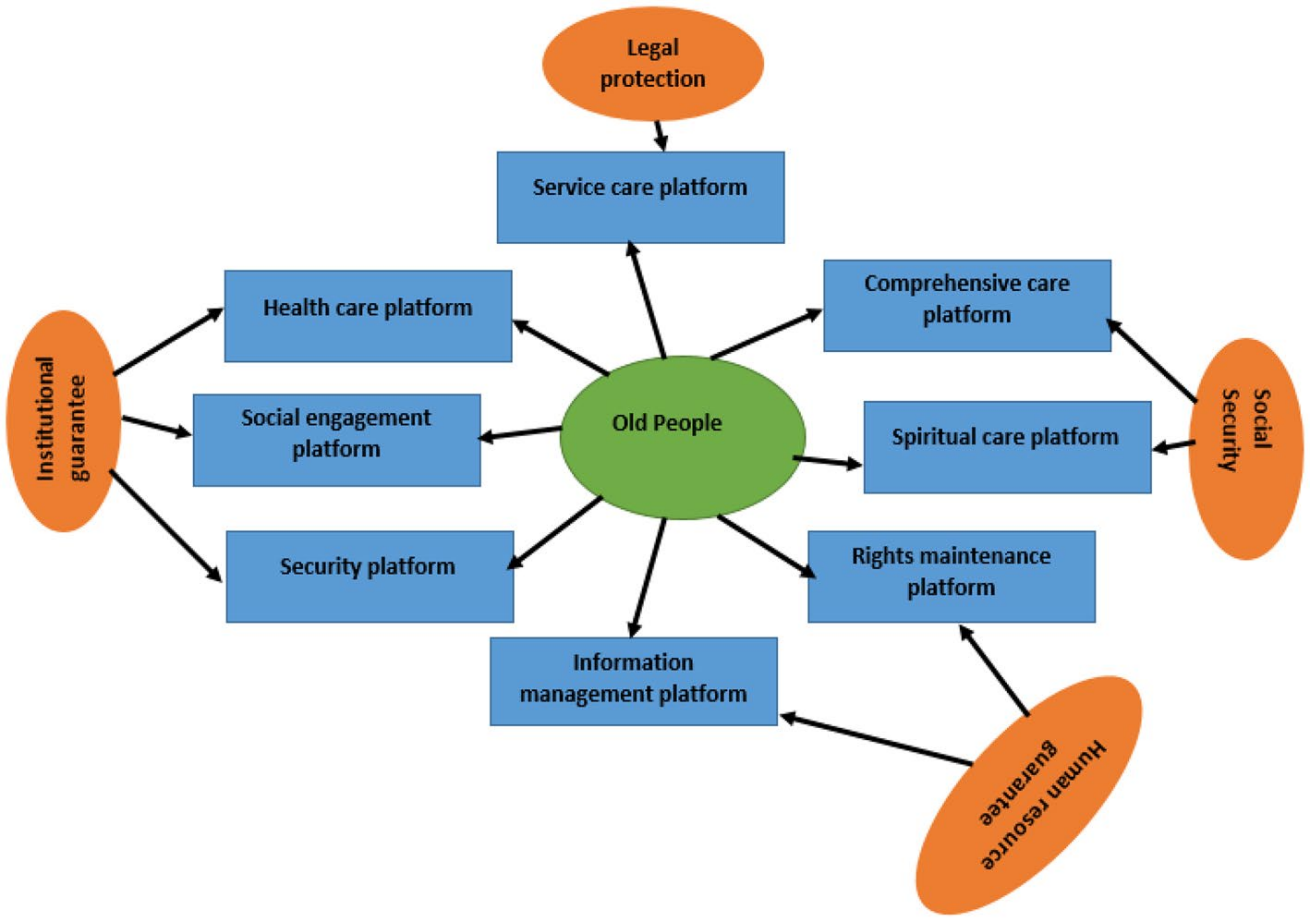
Security model of community elderly care service system.

In developing the ECS for the elderly in the community, it is imperative to consider the external objective environment of society and take into account its needs comprehensively. Therefore, multiple technical approaches can be employed to establish a community ECS system. It is essential to maintain a stable external environment and take an active and subjective approach to the development of the community's ECS system to form its unique characteristics. Figure 5 demonstrates the security model for the community pension service system.

Drawing upon the "Global Elderly Friendly City Construction Guidelines" promulgated by the World Health Organization (WHO) and taking into account the construction of the community Elderly Care Services (ECS) system, this paper posits that at least six technical approaches merit consideration. With respect to the living environment, it is imperative that the elderly are afforded a clean living space, with level flooring, clearly demarcated public facilities, and readily accessible restrooms and seating areas. Public safety must be guaranteed, and traffic planning should be rationalized.

In terms of living conditions for the elderly, it is imperative that the cost of living in their residential areas remains manageable. This includes considerations for essential utilities such as electricity, water, gas, as well as property fees. These financial aspects should align with the financial capacities of the elderly population. Additionally, housing structures and interior designs should be optimized for practicality, ensuring that they cater to the specific needs of the elderly. Basic amenities should be comprehensive, promoting ease of movement and independence for the elderly within their living spaces. Addressing social participation is equally crucial. Advocating for the notion of "doing something for the elderly and enjoying the elderly" is essential for fostering a sense of community and mutual support. This entails harnessing the enthusiasm of the elderly to actively participate in social activities. It involves recognizing and utilizing their unique skills and talents, encouraging their integration into various aspects of society, and providing platforms for them to offer valuable suggestions.

Social inclusion is a fundamental aspect that must be prioritized, particularly by reinforcing traditional Chinese filial piety values that emphasize respect and love for the elderly. Social policies should mirror this commitment, offering preferential treatment and prioritizing services tailored to the unique needs of the elderly population. Establishing robust community support is essential, involving well-trained service personnel equipped with both technical expertise and effective communication skills. Regular visits to the elderly are crucial, serving as a means to bridge the gap between them and society. These visits go beyond mere companionship; they also provide guidance in areas such as fitness, entertainment, learning, and socialization, addressing the holistic needs of the elderly and contributing to their self-realization.

In the realm of health services, community clinics play a pivotal role. Conducting regular health knowledge campaigns targeted at the elderly and organizing training sessions on topics like proper drug use and fitness science are essential components. Additionally, communities should invest in and expand rehabilitation and fitness training facilities to continually improve the health literacy of the elderly, promoting their overall well-being. By integrating these measures, communities can foster a more inclusive and supportive environment that not only respects traditional values but also addresses the multifaceted needs of the elderly population, promoting their health, happiness, and social engagement.

## Experiment and analysis of community elderly care service system

### Experimental design

The study centered on surveying individuals aged over 60 in area A. Out of 1000 distributed questionnaires, 897 responses were collected, of which 732 were validated, resulting in an 89.7% recovery rate and an 81.6% sampling efficiency. The key characteristics of the participants are outlined below:

#### Educational level of the elderly

**Figure 6:**
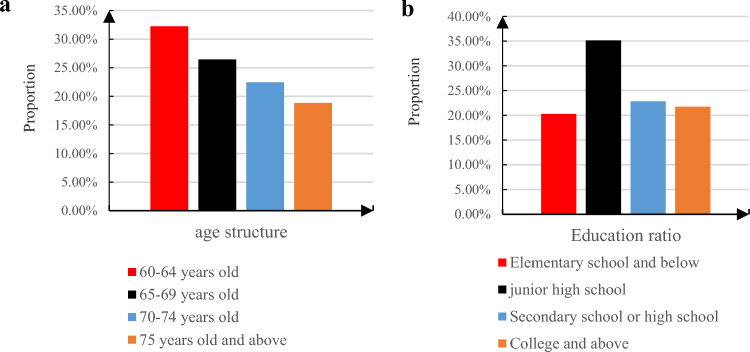
The proportion of respondents' age structure and the proportion of educational attainment. (**a**) The proportion of respondents’ age structure. (**b**) The proportion of respondents’ educational level.

The investigation specifically targets individuals aged 60 and above to assess the progress of intelligent elderly care. To bolster research validity, data obtained from this survey hold paramount significance. Age distribution and the educational background of respondents are visually presented in Fig. 6.

Drawing from interviews conducted with a representative subset of elderly individuals, the demographic profile reveals a well-balanced gender distribution, with males representing approximately 47% and females comprising around 53% of the sample. Across various age groups, the age distribution among elderly respondents appears to be evenly spread, indicating a diverse representation within the study. In terms of living arrangements, a slight majority of participants, totaling 41.67% of the sample, reported living either independently or with a spouse. Another significant portion, accounting for 39.13% of respondents, indicated cohabitation with their children. Conversely, alternative living arrangements were less common, making up only 19.2% of the total. Regarding educational background, there was a range of attainment levels among elderly participants. Notably, approximately 35% of the sample had completed junior high school education, while other education levels were distributed relatively evenly, each comprising around 21% of the total. These findings offer a comprehensive snapshot of the demographic characteristics observed among the interviewed elderly individuals. Insights into gender distribution, age composition, living arrangements, and educational backgrounds are valuable for gaining a nuanced understanding of the diverse needs and experiences within this demographic group.

**Figure 7:**
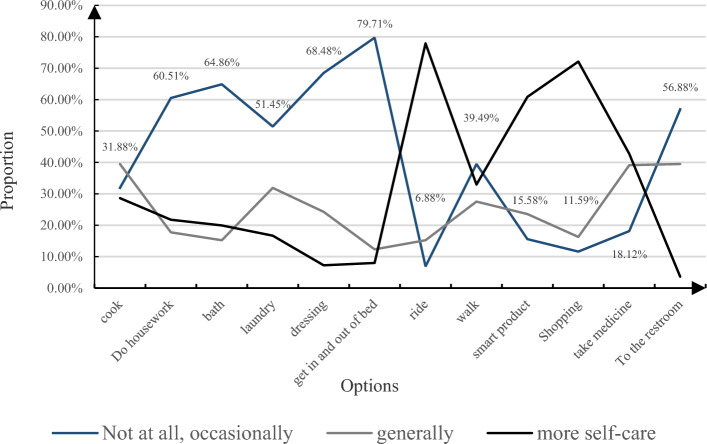
Statistics on the self-care of the elderly.

Regarding the self-care capability of the elderly, a pivotal aspect in understanding their social adaptability and demand for smart Elder Care Systems (ECS), an examination of their quality of life is essential. Investigating self-care habits and delving into their daily routines establishes a sturdy groundwork for crafting intelligent ECS content. This aspect's statistical dissection is portrayed in Fig. 7, offering insights into the breakdown of self-care behaviors among the studied elderly demographic.

As illustrated in Fig. 7, the examined elderly population displayed a generally robust daily living capability. Among the 12 self-care indicators, the top four scoring the highest were taking bus rides, utilizing smart products, shopping, and managing medications, with corresponding scores of 4.39, 3.33, 3.50, and 3.50, respectively. These outcomes imply the elderly's capacity to independently handle these tasks, even among those who might rely more on assistance. Consequently, it is crucial to factor in these tasks while formulating smart Elder Care Systems (ECS) solutions. Furthermore, the top five activities that respondents were able to perform autonomously and felt positive about were housework, bathing, dressing, moving in and out of bed, and using the toilet. Research indicates that most elderly individuals possess the ability to self-manage to some extent, albeit not necessarily indicating optimal health. As the elderly demographic ages, concerns regarding their physical well-being heighten. Hence, when crafting smart ECS solutions, consideration should be given to the elderly's physical condition, and ECS content should be tailored based on their self-care capabilities, particularly among those living alone, disabled, and semi-disabled individuals. For tasks like daily shopping and transportation within smart ECS setups, the elderly still necessitate data form of guidance and support from others.

### Respondents' understanding of smart elderly care services

**Figure 8:**
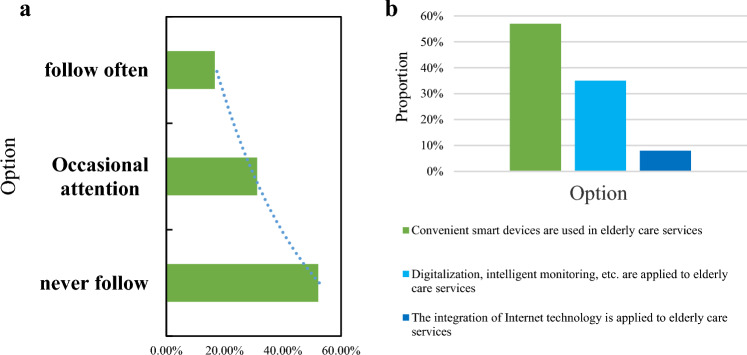
Percentage of respondents' understanding of smart old age care and smart old age care. (**a**) Proportion of respondents' attention to smart old-age care, (**b**) Proportion of respondents' understanding of smart old-age care.

Drawing upon the survey data, a fundamental comprehension of the elderly's perception of smart elderly care services emerges. By dissecting the attention devoted by respondents to smart elderly care and their grasp of this concept, an initial assessment can be conducted. The depiction of respondents' understanding of smart elderly care is presented quantitatively in Fig. 8.

The survey data reveals a significant trend: only 47% of elderly participants perceive "smart pension" as important, indicating a prevalent lack of awareness and limited importance attributed to this concept among the majority. Middle-aged and elderly respondents generally exhibit a pessimistic outlook on smart elderly care, with many possessing only a superficial understanding of its intricacies. Specifically, 57% perceive smart elderly care as the mere utilization of convenient smart products for elderly services, while a mere 8% demonstrate a deeper comprehension. These findings underscore a substantial knowledge gap among the elderly regarding smart old-age care, with educational attainment playing a role in shaping awareness levels. Notably, those with higher education levels exhibit greater awareness and openness to embracing new products emerging from advancements in internet technology. Recognizing this gap in knowledge, it becomes imperative to reshape perceptions of elderly care among those lacking sufficient understanding. Interviews with elderly individuals underscore the potential role of community workers in actively promoting smart old-age care. Encouraging the elderly to actively participate in the aging process, rather than being passive recipients of care, emerges as a pivotal strategy. By bridging the informational gap and cultivating a positive perspective, community workers can significantly contribute to enhancing awareness and fostering acceptance of smart old-age care within the elderly population.

### Respondents' demand for smart elderly care services

**Figure 9:**
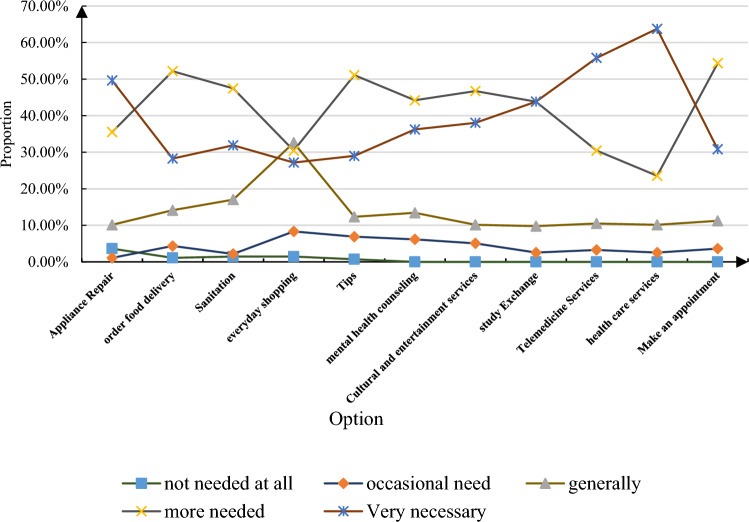
Demand for smart elderly care services for the elderly.

According to the selection of the elderly's demand for smart old-age care services, people can obtain information that is conducive to the enrichment and supplementation of smart old-age care services. When talking about what kind of help the elderly hope the smart ECS can provide, it was learned that elderly people with different physical conditions need different ECS. However, in general, it is hoped that smart ECS can provide life care services, spiritual comfort services and medical security services. After analyzing this, the demand for smart ECS for the elderly is shown in Fig. 9.

As shown in Fig. 9, it can be known that the needs of the elderly for smart ECS are more inclined to medical care services. At the same time, the elderly have a relatively high demand for home appliance maintenance, learning exchanges, telemedicine services, and health care services. At present, with the improvement of living conditions, the elderly are no longer simply seeking food and clothing when enjoying ECS but are more eager to meet high-level needs such as psychology, culture and entertainment. Moreover, most elderly people hope to apply smart devices reasonably and flexibly to ECS, and hope that more and better smart products can meet their elderly care needs. Therefore, in the provision of smart ECS, people should fully consider the elderly's elderly care needs, and "prescribe the right medicine" to make smart elderly care play its true meaning. When setting the content of smart ECS, services such as mental health consultation, cultural entertainment, telemedicine, and health care can be considered. The joining of offline service providers also needs to be screened according to the elderly's pension needs, to ensure the richness of service content and the reliability of service quality, so that the elderly can live a beautiful and colorful life in their later years.

### Respondents' acceptance of smart old age care

**Figure 10:**
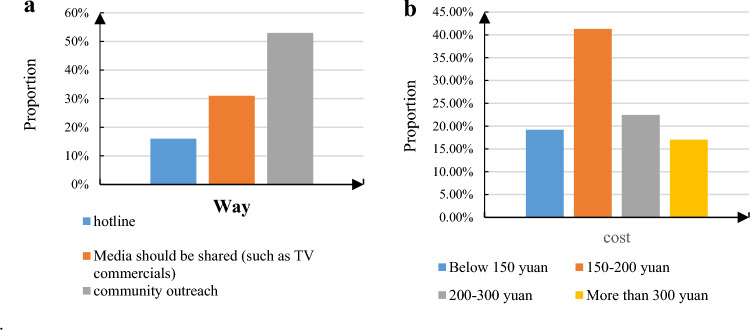
The way respondents obtain smart old-age care information and the proportion of smart old-age care service costs. (**a**) Proportion of access to smart old-age care information, (**b**) Proportion of smart old-age care service fees.

Based on the acceptance of smart old-age care among the elderly, the information channels for smart old-age care were explored, including the avenues through which elderly individuals access information, the provisioning of smart equipment, and the expenses elderly individuals are willing to incur for smart old-age care services. Initially, the questionnaire incorporated three methods to gather information about smart old-age care: a hotline, media-based promotion (such as television advertisements), and community-based awareness campaigns. Figure 10 illustrates the distribution of respondents' preferred methods for accessing information regarding smart old-age care and the percentage of costs they are willing to cover for smart old-age care services.

As shown in Fig. 10, most elderly people are willing to obtain information through community publicity channels, and only 16% choose the hotline to receive information on smart old age care. It can be seen that it is not desirable to use a hotline to provide information on smart ECS when promoting information on smart ECS. When promoting smart old-age care, people should try to choose a way that is more acceptable to the elderly for publicity, and the propaganda content should not only be text content, but can describe smart old-age care services with pictures and texts to achieve results. Second, several smart devices for the elderly to choose from were set up in the questionnaire. More than 85% of the elderly respondents want to have wearable monitoring devices, such as emergency pagers, GPS positioning bracelets, etc. There are also about 64% of the elderly who want to have intelligent sensing monitoring equipment, they think it can avoid their children worrying about themselves because they are not around for work. Another part of the elderly is worried that the intelligent sensing monitoring equipment would leak their privacy and affect their lives. Only 8% of the elderly accept intelligent robots, and the rest of the elderly think that intelligent robots are expensive and are less willing to accept intelligent robots. Therefore, at this stage, the elderly are more receptive to intelligent products with low prices, and the development of intelligent ECS should pay attention to the cost effectiveness of intelligent products.

At the same time, the following ranges are set for the costs of smart ECS: below 150 yuan, 150–200 yuan, 200–300 yuan, more than 300 yuan. Among them, the cost that most elderly people can afford is between 150 and 300 yuan, and there are not many elderly people who can afford more than 300 yuan. It can be seen that the smart ECS cannot cause the financial burden of the elderly when setting service fees. When providing intelligent equipment and ECS, the government should appropriately subsidize the elderly according to the age structure of the elderly.

### Respondents’ concerns about smart old age care

**Figure 11:**
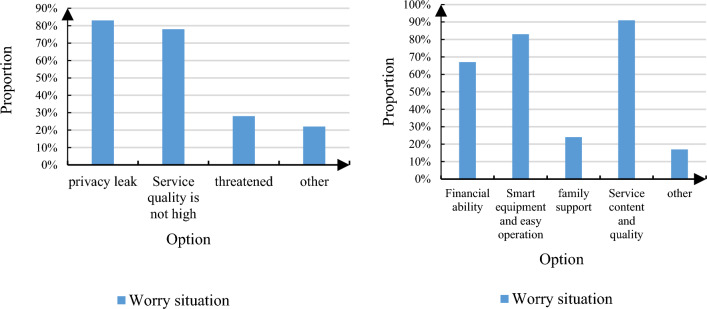
Respondents' concerns about smart old-age care and the proportion of factors that affect their choice of smart old-age care. (**a**) Proportion of concerns about smart old age care. (**b**) Proportion of factors influencing respondents’ choice of smart old age care.

A preliminary understanding of the concerns and concerns of the elderly about smart old-age care was carried out. As people all know, with the advent of the Internet era, people's lives are more and more convenient, and people's communication is also more and more convenient. This increases productivity and brings a range of benefits to lives. However, the development of Internet technology may have hidden dangers in network security, which may easily leak user privacy and cause unnecessary troubles. Smart old-age care itself needs to use the Internet, Internet of Things, big data and other information technologies to serve the old-age care industry, which would lead to the unsatisfactory acceptance of smart old-age care by the elderly. Figure 11 shows the proportion of respondents' concerns about smart old-age care and the factors that affect their choice of smart old-age care.

As depicted in Fig. 11, the survey findings reveal notable concerns among respondents regarding smart elderly care. A significant majority, approximately 83% of elderly participants, expressed worries about potential breaches of their privacy, indicating widespread apprehension within this demographic. Moreover, around 78% voiced concerns regarding the adequacy of service quality, underscoring the importance of ensuring high standards in the implementation of smart elderly care services. A smaller portion of elderly individuals expressed specific concerns, such as potential threats to personal property from door-to-door services offered by smart Elderly Care Service (ECS) personnel. Similarly, a minority cited anxieties about the accuracy of health data detection, recognizing its potential impact on medical treatment timelines or diagnosis accuracy. The survey also identified various influential factors shaping the development of smart elderly care. These factors include the financial capacity of the elderly, the user-friendliness of smart devices, familial support, and the content and quality of smart ECS offerings. Notably, over 90% of respondents regarded service content and quality as pivotal determinants in their considerations, highlighting the importance of delivering meaningful and high-quality services. Additionally, more than 83% considered the ease of operation of smart devices as a crucial factor influencing their decision-making, underscoring the significance of user-friendly technology in the adoption of smart elderly care solutions.

### Ethics approval and consent to participate

This study was approved by the Ethics Committee of Harbin University (approval number: HUB20230016), All participants were consented by an informed consent process that was reviewed by the Ethics Committee of Harbin University and certify that the study was performed in accordance with the ethical standards as laid down in the 1964 Declaration of Helsinki.

## Conclusions

The significance of research on innovative elderly care services lies in its potential to enhance the quality of life and social adaptation ability of older people. Based on the data analysis of 1000 respondents from Heilongjiang Province, this article has preliminary analyzed the self-care ability of older people and their understanding of intelligent elderly care. The initial analysis indicates that most elderly individuals can self-care. But lack understanding of smart elderly care. Those with higher education levels exhibit better comprehension and greater acceptance of new products. The study suggests that community workers should actively promote smart elderly care to alter perceptions of aging. However, the research acknowledges limitations, including a small sample size and geographical constraints, necessitating further studies. Recommendations include developing elderly-friendly smart products and addressing challenges in social elderly care services. The call for enhanced scientific research abilities aims to provide more accurate and credible conclusions in research endeavors. The findings show that older people can take care of themselves without relying too much on others, but they lack an understanding of smart elderly care. Older people with higher education levels have a better understanding of smart elderly care and are more likely to accept new products. Therefore, community workers should actively promote smart elderly care and change the concept of old-age care. However, the limitations of the research, including the small sample size and geographical limitations, must be addressed by advance studies. The research on innovative elderly care services holds tremendous promise in improving the quality of life and social adaptation abilities of older individuals. Drawing from the data analysis of 1000 respondents from Heilongjiang Province, this study highlights significant insights into the self-care abilities of older people and their understanding of intelligent elderly care. Moreover it is needed to develop smart products suitable for older people and address the challenges and constraints of social elderly care services. Additionally, future research should strive for a more robust scientific research ability to provide more accurate and credible conclusions.

## Data Availability

Enquiries about data availability should be directed to the corresponding author.
